# Supplementary material for: Empirical methods for controlling false positives and estimating confidence in ChIP-Seq peaks
Source: BMC Bioinformatics. 2008 Dec 5;9:523. doi: 10.1186/1471-2105-9-523 (PMC2628906; doi:10.1186/1471-2105-9-523)
Supplement: Additional File 1 — A variety of html documents from the USeq web site detailing the available applications, their best usage, output file type descriptions, command line menus, etc. [file 1471-2105-9-523-S1.zip › USeqUserGuides/JavaDoc.zip!/JavaDoc/trans/cel/ControlStats.html]

ControlStats


|  |  |  |
| --- | --- | --- |
| **Overview** \| **Package** \| **Class** \| **Use** \| **Tree** \| **Deprecated** \| **Index** \| **Help** | |  |
| **PREV CLASS**   **NEXT CLASS** | **FRAMES**    **NO FRAMES**     **All Classes** |  |
| SUMMARY: NESTED \| FIELD \| CONSTR \| METHOD | DETAIL: FIELD \| CONSTR \| METHOD |  |


---


## trans.cel Class ControlStats

```
java.lang.Object
  trans.cel.ControlStats
```

**All Implemented Interfaces:**: java.io.Serializable

---

``` public class ControlStats extends java.lang.Object implements java.io.Serializable ```

**See Also:**: Serialized Form

---

**Constructor Summary**

`ControlStats()`


| **Method Summary** | |
| --- | --- |
| `java.lang.String[]` | `getNames()` |
| `double[]` | `getStats()` |
| `boolean` | `ordered()` |
| `void` | `setNames(java.lang.String[] names)` |
| `void` | `setStats(double[] stats)` |
| `java.lang.String` | `toString()` |

**Methods inherited from class java.lang.Object**

`equals, getClass, hashCode, notify, notifyAll, wait, wait, wait`

**Constructor Detail**

### ControlStats

```
public ControlStats()
```


**Method Detail**

### ordered

```
public boolean ordered()
```

---


### toString

```
public java.lang.String toString()
```

:   **Overrides:**: `toString` in class `java.lang.Object`

---


### getNames

```
public java.lang.String[] getNames()
```

---


### setNames

```
public void setNames(java.lang.String[] names)
```

---


### getStats

```
public double[] getStats()
```

---


### setStats

```
public void setStats(double[] stats)
```


---


|  |  |  |
| --- | --- | --- |
| **Overview** \| **Package** \| **Class** \| **Use** \| **Tree** \| **Deprecated** \| **Index** \| **Help** | |  |
| **PREV CLASS**   **NEXT CLASS** | **FRAMES**    **NO FRAMES**     **All Classes** |  |
| SUMMARY: NESTED \| FIELD \| CONSTR \| METHOD | DETAIL: FIELD \| CONSTR \| METHOD |  |


---
